# An Fe(III)-Based Fluorescent Probe for Carbon Monoxide only Senses the “CO Donor” Used, CORM-3, but Not CO

**DOI:** 10.1021/acs.analchem.5c04712

**Published:** 2025-10-09

**Authors:** Hongliang Li, Dongning Liu, Binghe Wang

**Affiliations:** Department of Chemistry and Center for Diagnostics and Therapeutics, 1373 Georgia State University, Atlanta, Georgia 30303, United States

## Abstract

Because of the increasing interests in carbon monoxide (CO) as an endogenous signaling molecule, there have been extensive efforts in developing fluorescent probes for CO. In doing so, metal–carbonyl complexes named “CO-releasing molecules” (CORMs) are often used as CO surrogates. The most widely used CORM-2 and CORM-3 are chemically reactive Ru­(II) complexes; release minimal or no CO unless in the presence of a strong nucleophile or a reducing agent; and do not function as reliable CO donors. As a result, some reported CO fluorescent probes only detect the CORM used, not CO. Recently, an Fe­(III)-fluorophore complex, RBF-Fe­(III), has been reported to sense CO using CORM-3 as a CO surrogate. The proposed mechanism involves CO binding to Fe­(III). Because of the known affinity of CO for only Fe­(II), but not Fe­(III), we were intrigued by the report. Re-evaluation work found fluorescence changes of RBF-Fe­(III) by CORM-3, but not CO itself. Furthermore, sodium ascorbate and cysteine were found to induce fluorescent changes of the RBF-Fe­(III) system. Moreover, RBF-Fe­(III) was found to be unstable and to change fluorescence with time or agitation. Regardless of whether it was under N_2_, CO, or vacuum, vigorous stirring induced the same level of fluorescence changes, presumably due to precipitation or aggregation of Fe­(III) species, which is consistent with literature findings of Fe­(III) behaviors. Such results mean that the RBF-Fe­(III) system does not sense CO and underscore the need to exercise extra cautions when chemically reactive CO donors are used in developing CO probes.

## Graphical abstract

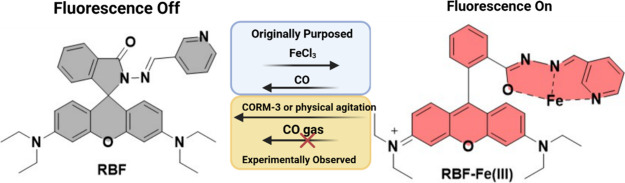

## Introduction

There has been an increasing level of interest in studying carbon monoxide (CO) because of its demonstrated potential as a therapeutic agent and its proposed endogenous production.^1,2^ As a result, there remains a need for research tools, such as optical probes, for studying CO biology. Along these lines, there has been highly innovative work in developing reaction-based fluorescent probes for CO to aid in the study of CO biology and pharmacology.^3^ These designs are based on the creative use of known CO chemistry, including Pd-mediated carbonylation of various types^3–5^ and CO’s ability to reduce Pd­(II) to Pd(0),^6,7^ which catalyzes deallylation from an ether or ester moiety.^8,9^ Along a similar line, we have also reported a reaction-based CO probe through de novo-construction of a fluorophore via Pd-mediated carbonylation.^5^ Though, there has been tremendous success in this area, there are also many problems in this field largely because of the use of chemically reactive CO donors, which are carbonyl complexes with chemically reactive moieties such as BH_3_ or metal ions such as Ru­(II) and Mn­(I). There are four commercially available donors named as CO-releasing molecules (CORMs) including CORM-2, CORM-3, CORM-401, and CORM-A1.^10^ The use of these chemically reactive CORMs has led to reports of “CO probes” that only respond to the presence of the CORM used as “CO surrogate,” but not CO itself. Essentially, all such “CO probes” rely on “CO chemistry” that had never been observed before their reports in developing fluorescent CO probes. For example, CO was said to reduce an aromatic nitro group to an amino group under near-physiological conditions to achieve “CO sensing.” Such reports contradict known chemistry of CO and aromatic nitro groups, which require strongly reductive conditions for conversion to an amino group, such as catalytic hydrogenation, metal-acid mixtures, or reductive metal ions. Many of these chemistry issues have been discussed in depth in a recent *Perspective* paper.^11^ Beyond all the issues already discussed, there is a recent report of using an Fe­(III)-based “strategy for detecting carbon monoxide.”^12^ Specifically, a latent fluorophore RBF was said to bind to Fe­(III), leading to a fluorescent complex RBF-Fe­(III). Addition of CO was said to bind to Fe­(III) and dissociate the complex to give nonfluorescent RBF (Scheme 1). The design was said to be based on heme iron’s “pronounced propensity for binding with CO.” However, the chemistry is such that only the ferrous/Fe­(II) form binds to CO (K_a_ is on the order of 10^6^–10^8^ M^–1^),^13^ but not the ferric/Fe­(III) form. The tight binding with ferrous iron has a special reason because of π-back bonding, which does not exist in the ferric form.^14–16^ Furthermore, the study employed CORM-3 as a CO surrogate, despite its well-known chemical reactivity. This reported ability of an Fe­(III)-based probe to detect CO was therefore intriguing, prompting us to investigate and seek clarification on this issue.

**Scheme 1:**
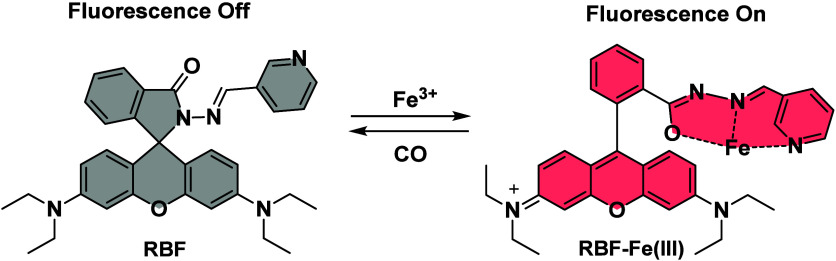
Schematic Representation of the Originally Proposed Sensing Mechanism for the Fe­(III)-Based Fluorescent CO Probe RBF-Fe­(III)

## Experimental Section

### Material and Instruments

Chemical reagents were purchased from Sigma-Aldrich (Saint Louis, MO) and/or Oakwood (Estill, SC). Solvents were purchased from Fisher Scientific (Pittsburgh, PA). Dry solvents were prepared by a Vigor Tech purification system (Houston, TX). Certified pure CO calibration gas was purchased from GASCO (Oldsmar, FL). UV–vis absorption spectra were obtained by using a Shimadzu PharmaSpec UV-1700 UV–visible spectrophotometer (Kyoto, Japan). Fluorescence spectra were recorded on a Shimadzu RF5301PC fluorometer (Kyoto, Japan). ^1^H NMR (400 MHz) and ^13^C NMR (101 MHz) were acquired on a Bruker AV-400 MHz Ultra Shield NMR.

#### Synthesis of the RBF

RBF was synthesized following the literature procedure.^12^ Detailed procedures and compound characterizations are described in the Supporting Information file.

#### Stock Solution Preparation

Following literature procedures, RBF stock solution was prepared in DMSO at a concentration of 1 mM. The FeCl_3_ stock solution (1 mM) was prepared by dissolving ferric chloride (FeCl_3_) in deionized water. All RBF solutions for spectroscopic experiments were prepared in a DMSO/H_2_O solution (v/v = 1:4) with a final concentration of 10 μM. As an example, 750 μL of deionized water, 190 μL of DMSO, 50 μL of FeCl_3_ stock solution (1 mM) and 10 μL of RBF stock solution (1 mM) were added to a 1.5 mL cuvette to get a 10 μM RBF-Fe solution.

#### Spectroscopic Experiments of RBF

UV and fluorescence experiments of RBF (10 μM, DMSO/H_2_O solution (v/v = 1:4)) were carried out at room temperature. The fluorometer instrument parameters were set as λ_ex_ = 450 nm, 5.0 nm excitation slit width, 5.0 nm emission slit width, and high sensitivity of detection. All experiments were done in triplicate.

#### Effects of CORM-3 on the Fluorescence of RBF

CORM-3 in deionized water or DMSO were prepared as 10 mM stock solutions. As an example, 0.59 mg of CORM-3 was weighed by a microbalance. Then, 201 μL of deionized water was added to get a 10 mM CORM-3 stock solution. For the Fe^3+^ experiments, after preparing a 10-μM RBF solution in 1.5 mL cuvettes, 50 μL (50 equiv) of Fe^3+^ stock solution (10 mM) was added to the cuvette followed by mixing via pipetting and releasing. For the effects of CORM-3 on RBF, 8 μL (6 equiv), 16 μL (12 equiv), 24 μL (18 equiv), 32 μL (24 equiv) or 40 μL (30 equiv) of CORM-3 stock solution (10 mM) was added to the RBF- Fe­(III) solution (10 μM each) respectively followed by mixing via pipetting and releasing.

#### Effects of CO Gas on the Fluorescence of RBF

For the effects of CO gas on the fluorescence of RBF, CO gas was directly bubbled into the RBF-Fe­(III) solution (both 10 μM) for 15 min.

## Results and Discussion

### Synthesis and Structural Confirmation of RBF

In the original publication, the key latent fluorophore used was RBF (Scheme S1). As the first step of the validation work, we synthesized RBF following the reported literature procedure.^12^ The product was characterized by using mass spectrometry, ^1^H- and ^13^C NMR (Figures S1–S3) through comparison with literature data (Figures S4–S6). Our work confirms the structure of RBF as stated in the original publication.

### Confirmation of Literature Findings in Sensing CORM-3

Before we assessed the probe’s ability to sense CO using a pure CO source, we were interested in confirming literature findings of the probe’s spectroscopic properties and its response to CORM-3 as described in the original publication. As shown in Figure 1, addition of FeCl_3_ to RBF in DMSO/H_2_O (v/v = 1:4) indeed induced a significant fluorescence intensity increase, in agreement with that of the original publication. Such results serve as secondary validation of the literature findings on Fe­(III)’s effect on the fluorescence of RBF.

**Figure 1:**
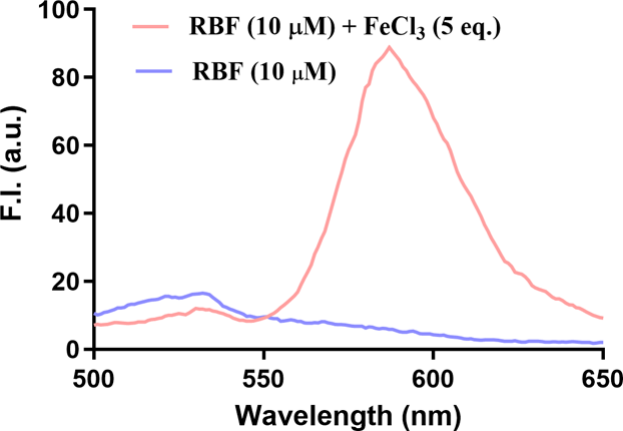
Fluorescence emission spectra of RBF (10 μM) and RBF-Fe­(III) (10 μM RBF + 50 μM FeCl_3_) in DMSO/H_2_O (v/v = 1:4). Upon addition of FeCl_3_, a distinct emission peak emerges at 590 nm, indicating formation of the fluorescent RBF-Fe complex­(III) as suggested by the original publication (Ex= 450 nm, slit width = 5 nm).

Next, we compared its UV–Vis absorption response of RBF to FeCl_3_ and CuSO_4_, both at 5 equiv. As shown in Figure 2, addition of FeCl_3_ to RBF (10 μM) resulted in a distinct increase in absorbance at ∼564 nm. However, addition of CuSO_4_ only led to minor changes with a small peak at 564 nm. Such results qualitatively agree with the preferential binding of RBF with Fe^3^^+^ over Cu^2^^+^, consistent with the original report (Figure S7).^12^ Fe­(NO_3_)_3_ also led to similar UV–vis spectral changes as that of FeCl_3_ (Figure S8), indicating the general effect of Fe­(III) regardless of the counterion (Cl^–^ or NO_3_^–^) used. Interestingly, for fluorescence studies in the original publication, excitation wavelength was set at 450 nm while there is no visible UV peak at this wavelength (Figure 2). We conducted an excitation spectral scan (Figure S9) and found the λ_ex_ to be around 570 nm, not 450 nm. In fact, 450 nm seems to be the overall minimum in the excitation spectrum, leading to a scenario of least sensitivity. We are uncertain why the original publication selected this least-sensitive wavelength for analytical work. However, to enable direct comparison with the results from the original study, we used 450 nm as the excitation wavelength in our subsequent experiments.

**Figure 2:**
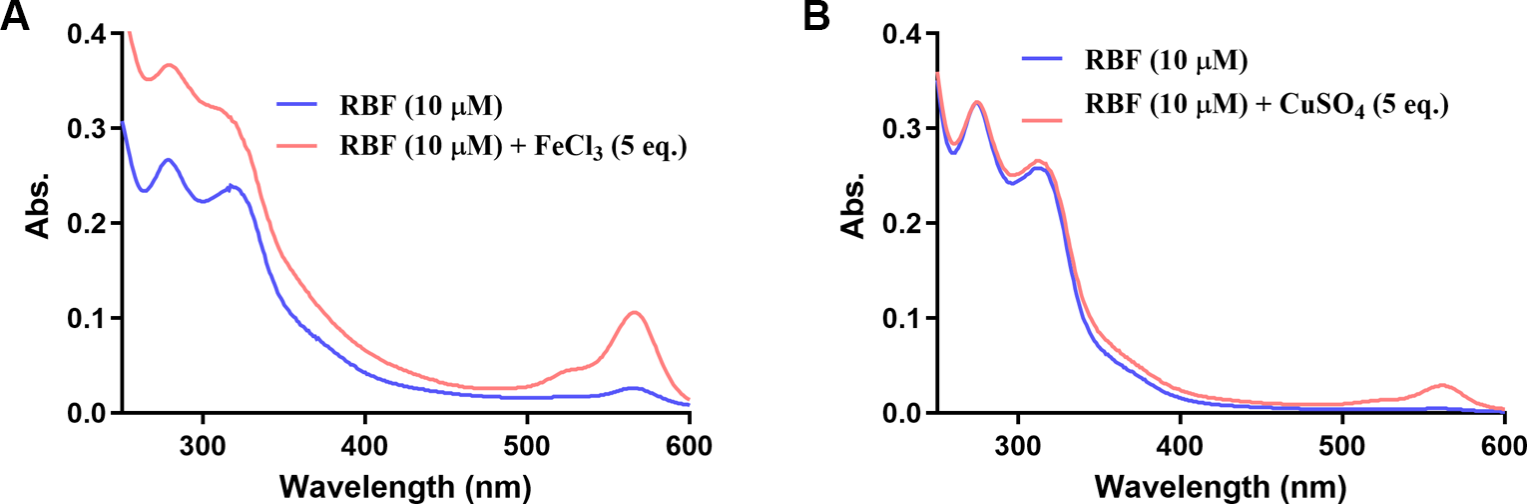
UV–Vis absorption spectra of RBF (10 μM) after the addition of FeCl_3_ (A) or CuSO_4_ (B), respectively. A characteristic absorbance peak appears around 564 nm upon the addition of FeCl_3_.

Then, we examined the concentration dependence of RBF’s responses of FeCl_3_. As shown in Figure 3, adding Fe^3+^ to 10 μM RBF solution (DMSO/H_2_O, v/v = 1:4) led to increase in fluorescent intensity in a concentration-dependent fashion as described in the original publication. The relationship seems to be linear in the region of 20–50 μM, a clear indication of a presaturation phase and weak affinity. The observed effect of Fe­(III) on RBF fluorescence was proposed to be due to the chelation effect of Fe­(III) as shown in Scheme 1. Though there is no dispute of the experimental findings, we do not agree with the RBF-Fe­(III) structure as drawn in the original publication. This is because RBF was shown to have three coordination bonds with Fe­(III) (Scheme 1) involving two nitrogen atoms and one oxygen atom all in *sp* ^2^ hybridization, affording a geometry unlikely to be consistent with the widely known Fe­(III) geometry (octahedral or tetrahedral). Furthermore, a 6-membered ring involving pyridine nitrogen in a “trans-like” geometry is expected to be severely (or impractically) strained. Nevertheless, our experimental findings are consistent with what were presented in the original study.

**Figure 3:**
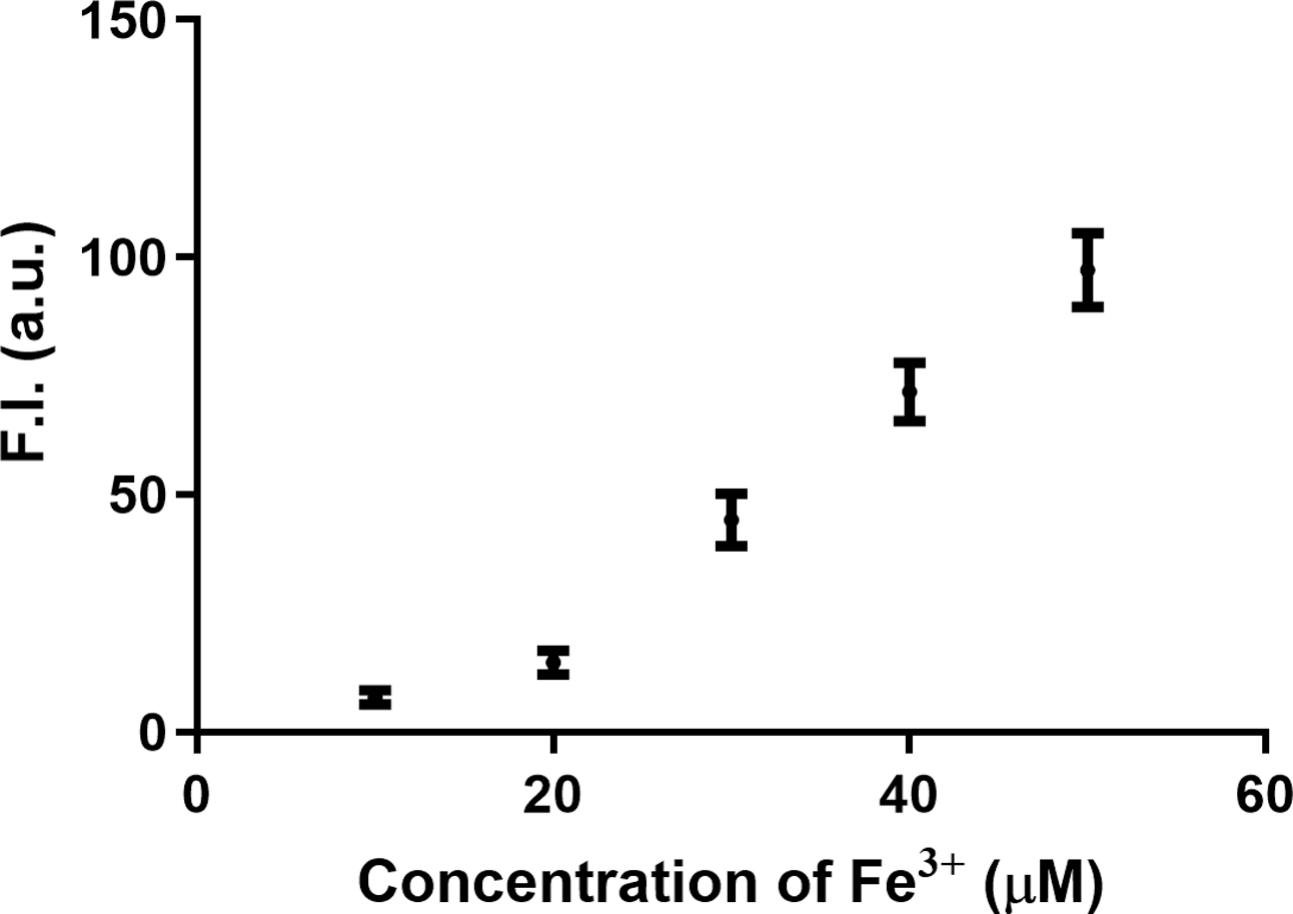
Concentration-dependent effects of Fe^3+^ (1, 2, 3, 4, and 5 equiv) on the fluorescence of RBF solution (10 μM) in DMSO/H_2_O (v/v = 1:4). (Ex = 450 nm, λ_em_ = 590 nm, slit width = 5 nm, *n* = 3, and mean ± SD).

Next, we examined the spectroscopic response of the RBF-Fe­(III) solution to CORM-3 in DMSO, as described in the original publication. Specifically, CORM-3 was initially dissolved in DMSO to prepare a stock solution and then diluted into the aqueous RBF-Fe­(III) solution (10 μM) to final concentrations of 60, 120, 180, 240, or 300 μM. The addition of CORM-3 led to significant fluorescence intensity changes, as shown in Figure 4A. While our results showed a general agreement with the original publication regarding the ability of RBF-Fe­(III) to detect CORM-3 in DMSO, we did not observe the same concentration dependency as reported by the original publication (Figure 4A and S10 vs S11a). The original studies showed a linear concentration dependency (Figure S11a) in the concentration range of 0–300 μM of CORM-3, while we saw idiosyncratic fluorescence intensity fluctuations in the region of 60–300 μM (Figures 4A and S10). We should note that CORM-3 stability problems in DMSO have been long established through extensive studies.^10,17,18^ Further, CORM-3 in DMSO is known to lead to rapid CO release within mins. During the experiments (Figure S10), color changes of the CORM-3 stock solution were observed. The color was initially yellow upon preparation but gradually faded to become completely transparent within approximately 2 h. Such color changes are consistent with the known stability problem of CORM-3 in DMSO,^17,19,20^ which could lead to significant variations in its interaction with the RBF-Fe complex and contribute to the nonlinear fluorescence response that we observed. Further, the DMSO-mediate CO release from the CORM-3 stock solution creates an intractable situation if the goal was to detect CO even semiquantitatively.

**Figure 4:**
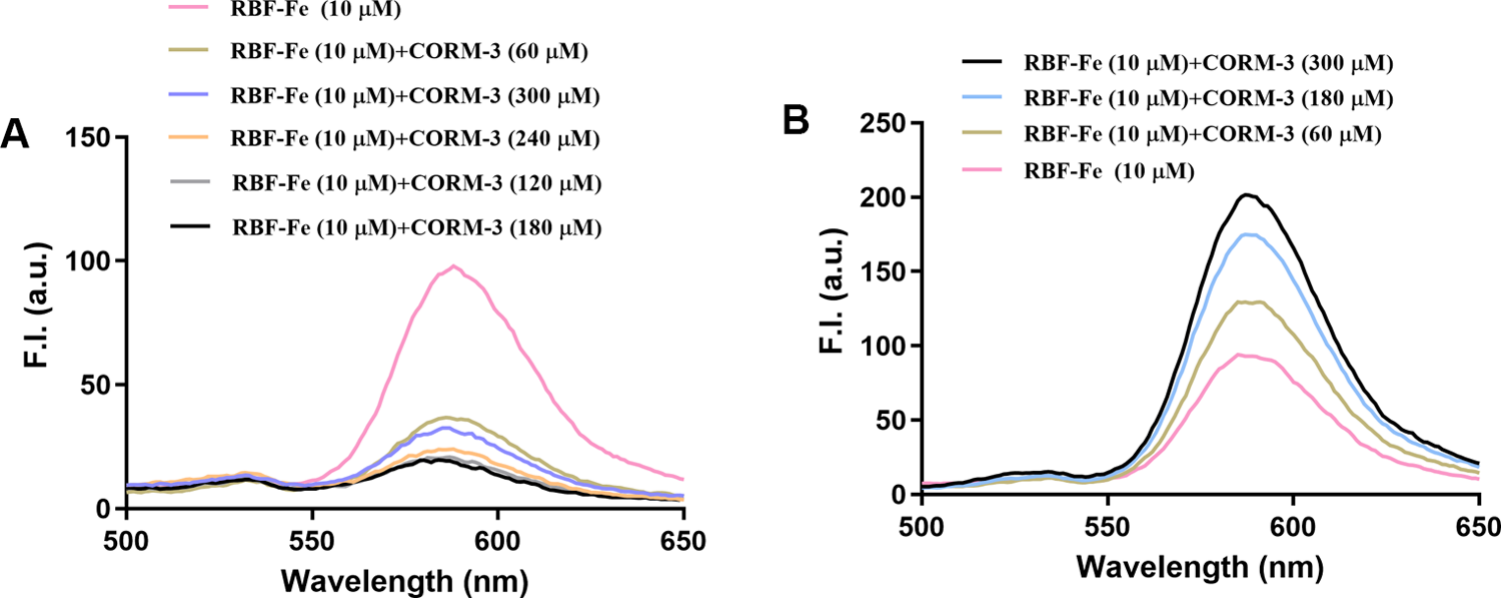
Effects of CORM-3 (60–300 μM in H_2_O) on the fluorescence of RBF-Fe complex (RBF-10 μM, FeCl_3_ 50 μM) in DMSO/H_2_O (v/v = 1:4). (A) CORM-3 stock solution was prepared in pure DMSO, according to the original literature. (B) CORM-3 stock solution was prepared in pure water. (Ex = 450 nm, λ_em_ = 590 nm, and slit width = 5 nm).

NMR was used to study Fe­(III)–rhodamine coordination. Because of the importance of such experiments, we also performed ^1^H NMR experiments. There are two aspects to the experiments. First, the original paper conducted NMR experiments using a 400 MHz instrument at 10 μM,^12^ which is way below what is normally used in acquiring NMR data for such small organic molecules. Indeed, when we did our experiments at the same concentration, we did not see any meaningful signals after overnight acquisition on a 400 MHz instrument and therefore were unable to duplicate what was reported. Second, we conducted similar experiments at 1 mM (Figure S15) rather than 10 μM.^12^ Under such conditions, we only observed the hydrazone proton upfield from 9.12 to 8.97 ppm. As expected, increasing FeCl_3_ concentration in the solution led to peak broadening because of the Fe­(III)’s paramagnetic nature.^21,22^ Further, we did not observe the same spectral features consistent with the Fe­(III)–rhodamine complex as proposed because one would expect the complexed ring-open form to be quite different from that of the closed form in NMR (Scheme 1).

Overall, the results described above are largely consistent with literature findings in terms of the probe’s spectroscopic properties, fluorescence enhancement by Fe^3+^, and the ability for CORM-3 to decrease the fluorescence of the RFB-Fe­(III) solution. However, these results should not—and cannot—be interpreted as evidence of CO sensing. Furthermore, we did not observe a linear concentration-dependent effect of CORM-3 on the fluorescence of the RBF-Fe­(III) complex and were unable to duplicate the NMR experiments.

### New Findings about RBF-Fe­(III) Using CORM-3 in H_2_O

The solvent environment plays a critical role in determining the behavior of CORM-3 and its ability to modulate fluorescence.^10,17,23^ For applications in studying CO biology, it is important to stay with aqueous solutions as much as possible. Therefore, we also conducted experiments using CORM-3 dissolved in water. Furthermore, CORM-3 is known to be more stable in an aqueous solution than in DMSO, which can quickly lead to CORM-3 degradation.^10,17,18^ While fluorescence decrease was observed using CORM-3 prepared in a DMSO stock solution, adding CORM-3 prepared in an aqueous stock solution led to fluorescence enhancement of the RBF-Fe­(III) solution (Figure 4B). CORM-3′s sensitivity to solvent also suggests difficulties in conducting experiments in a controllable fashion if the detection system is in cell culture.

Additionally, we would like to add one more observation to this discussion. In 2024, we reassessed two CO probes (RCO and DEB-CO), which are also hydrazone-based compounds (structurally similar to RBF).^24^ In the 2024 study, addition of CORM-3 prepared in a PBS stock solution led to an increase in the fluorescence intensity (590 nm) of these two probes, in agreement with the result in Figure 4B, providing literature precedents of similar observations.

### New Findings about RBF-Fe­(III) Using CO gas

To study whether a probe truly detects CO, it is critical to include experiments using a pure CO source. It should be noted that in the original publication, the RFB-Fe­(III) system was studied upon exposure to CO gas for 0–18 min. Because there was no detailed description of the CO exposure experiments, we used a routine method in our lab that has been shown to give high micromolar concentrations of CO in solutuon.^5,25,26^ Specifically, pure CO gas (10 psi) was gently bubbled directly into the RBF-Fe­(III) solution in a cuvette through a long syringe needle (Figure S12). After 18 min of bubbling, the fluorescence data were recorded and compared with the control group. As shown in Figure 5, no fluorescence change was observed upon exposure to CO gas. As expected, control experiments using N_2_ gas also did not alter the fluorescence of the system. The results are unambiguous: RBF-Fe (III) did not response to CO at high micromolar concentrations. This is in direct contrast to what was described in the original publication (Figure S11b), which showed linear and time-dependent responses to CO.

**Figure 5:**
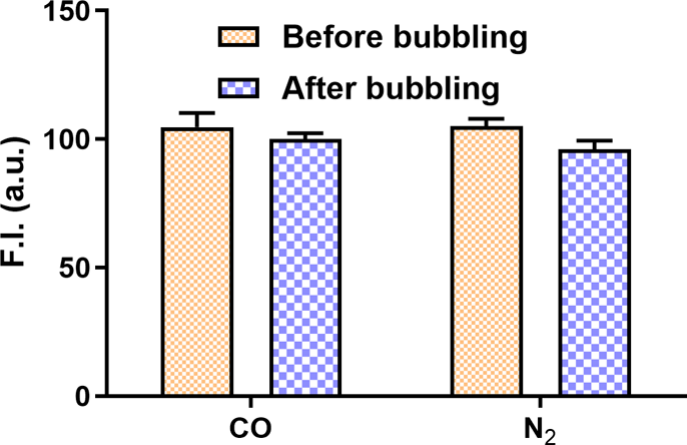
Fluorescence intensity changes of RBF-Fe­(III) complex (RBF-10 μM, FeCl_3_ 50 μM) after bubbling CO or N_2_ gas for 18 min. (Ex = 450 nm, λ_em_ = 590 nm, slit width = 5 nm, *n* = 3, and mean ± SD).

At this point, we reached out to the authors of the original publication for a detailed procedure for the CO exposure experiments to make sure that we were making a valid comparison. The procedure we received indeed was different from what we used to generate the data in Figure 5. Specifically, the original procedure involved vigorous stirring of the RBF-Fe­(III) solution upon exposure to CO. Following the procedures provided by the original authors, the RBF-Fe­(III) solution was prepared in a round-bottom flask. Then air was evacuated using vacuum before a pure CO balloon was connected to the flask followed by stirring at 800 rpm for 18 min at room temperature. Such a procedure indeed led to a significant fluorescence intensity decrease (Figure 6A), which is qualitatively consistent with the data reported. The only difference was time dependency. We saw the same result at both 1 min and 18 min time points (Figure 6A), while the original publication (Figure S11b) described a near-perfect linear relationship with time. Puzzled by the significant difference between bubbling CO (Figure 5) and exposing the RFB-Fe­(III) solution to a CO balloon followed by stirring, we made efforts to examine the effects of vigorous stirring on the RFB-Fe­(III) solution. Specifically, we examined the effects of stirring under N_2_ or after evacuation of the air in the system (Figure 6B,C). As one can see from Figure 6, the fluorescence intensity of the RBF-Fe­(III) complex upon stirring under CO, N_2_ or under vacuum was found to be the same in magnitude. Figure 6D puts all the data in one bar graph for easy comparisons. Such results indicate that mechanical stirring was the cause of fluorescence intensity change, not exposure to CO. The instability of Fe­(III) salt in aqueous solution has been very well-established. Hydrolysis can lead to polymer formation and precipitation among other things. In a 1984 publication in *Chemical Reviews,* Flynn comprehensively discussed the issue of Fe­(III) stability, collectively considered part of an aging process including hydrolysis and precipitation of various Fe­(III) species in aqueous solutions.^27,28^ Many factors are known to affect the process including pH, counterion, temperature, and centrifugation, among others. We reasoned that mechanical stirring accelerates the aging process of FeCl_3_ in the RBF-Fe solution and leads to the dissociation between the RBF and Fe­(III), resulting in the fluorescence intensity decrease observed. All the evidence points to the conclusion that fluorescence changes are not attributable to CO, regardless of the detailed mechanism, which is a complex topic as shown by the extensive Fe­(III) aging literature. As such, this is not a CO sensing system.

**Figure 6:**
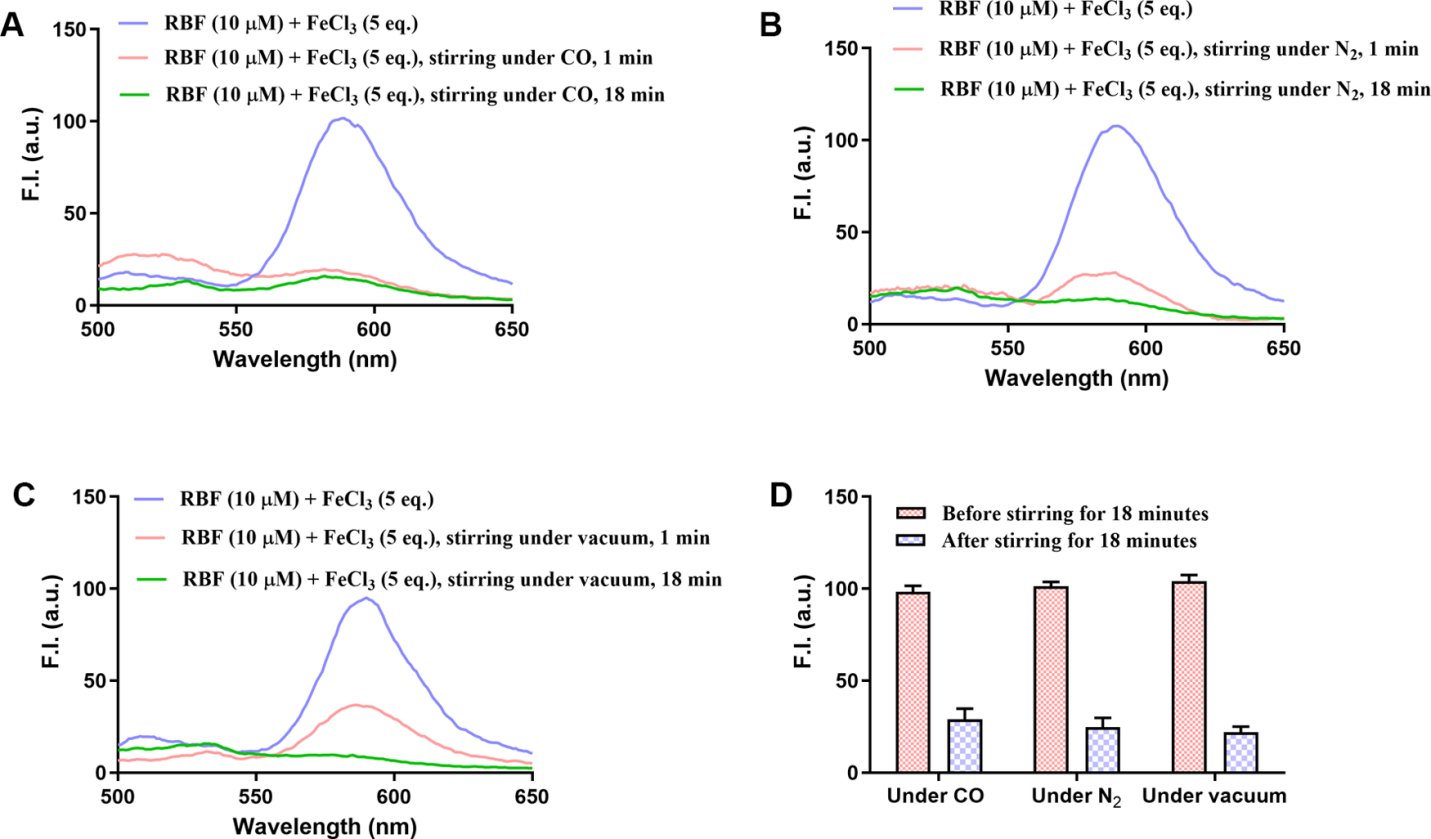
Fluorescence spectra of RBF-Fe complex (RBF-10 μM, FeCl_3_ 50 μM) after stirring at 800 rpm under different conditions for 18 min at room temperature. (A) Under pure CO gas, (B) under N_2_ gas, (C) under vacuum, and (D) fluorescence intensity changes at 590 nm of the RBF-Fe­(III) complex (10 μM) before and after 18 min of stirring under different conditions: CO, N_2_, and vacuum (Ex = 450 nm, λ_em_ = 590 nm, slit width = 5 nm, *n* = 3, and mean ± SD).

To further evaluate the stability of the RBF-Fe­(III) complex and its sensitivity to physical perturbations, we performed a time-course fluorescence study and a comparative analysis under various environments. As shown in Figure S13B, the fluorescence intensity of the RBF-Fe­(III) complex (10 μM) initially increased and then gradually reached a plateau within approximately 900 s under ambient conditions. However, upon vortex-mixing for 30 s at the 1000-s time point, a sharp drop in fluorescence was observed, followed by a new, stable fluorescence baseline at a significantly lower intensity. This abrupt change again indicates that the fluorescence signal of the complex is sensitive to physical agitation and is consistent with the results in Figure 6.

### New Findings about This Probe Using Sodium Ascorbate and l-Cysteine

To further investigate the sensitivity of the RBF-Fe­(III) complex, we introduced a biologically relevant reducing agent, sodium ascorbate (vitamin C or VcNa), and l-cysteine into the RBF-Fe­(III) system under controlled conditions (VcNa and l-cysteine were added gently without vigorous mixing). Specifically, in the presence of 30 or 60 μM VcNa, the RBF-Fe­(III) solution (10 μM) was incubated at room temperature, and fluorescence intensities were recorded after 30 min. As shown in Figure 7A, the presence of 30 μM VcNa caused a significant reduction in fluorescence compared to the control. Furthermore, increasing the concentration to 60 μM led to a further decrease in fluorescence intensity. For l-cysteine, 30 and 60 μM also led to fluorescent intensity decreases (Figure 7B). We also found that vortexing can further decrease the fluorescence intensity (Figure S14A). These results are different from that of the original publication, which used VcNa and cysteine as negative controls with no effect on the fluorescence of RBF-Fe­(III). We also observed a decrease in fluorescence intensity upon treatment with sodium acetate (Figure S14B). These results indicate that multiple factors can influence the performance of the RBF-Fe­(III) system, rendering it ineffective for CO detection.^29^

**Figure 7:**
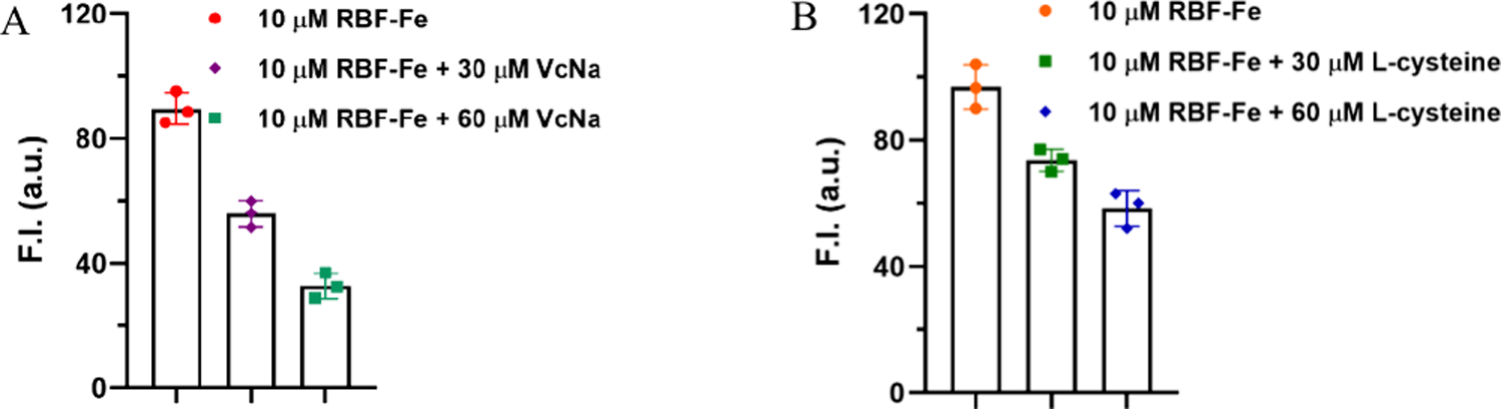
Fluorescence intensity changes of RBF-Fe complex (RBF-10 μM, FeCl_3_ 50 μM) after addition of (A) sodium ascorbate (VcNa) or (B) l-cysteine with a final concentration of 30 and 60 μM. Measurements were performed in a DMSO/H_2_O solvent system (v/v = 1:4). (Ex = 450 nm, λ_em_ = 590 nm, slit width = 5 nm, *n* = 3, and mean ± SD).

Taken together, these findings indicate that vigorous physical agitation can lead to substantial fluorescence loss in the RBF-Fe­(III) system. The fluorescence decreases of RBF-Fe­(III) observed using CORM-3 in DMSO cannot be attributed to CO release but rather likely by other reactive species associated with CORM-3 decomposition. Such results are consistent with literature reports of the affinity of CO only for Fe­(II), but not Fe­(III). These results highlight the need for careful attention to the underlying chemistry principles in CO detection and choice of CO donors used. Known chemical reactivities in CO donors such as CORM-2, CORM-3, and CORM-401 should be carefully considered before being used in a given experiment. Furthermore, experimental discussions should not use CORM and CO interchangeably because they are completely different chemical entities. Just because something is labeled a “CO-releasing molecule” does not necessarily mean it actually releases CO or functions effectively as a CO surrogate.

## Conclusion

In this study, we re-evaluated CO sensing by a Fe­(III)-based fluorescent probe, RBF-Fe­(III). Our experimental findings only support the ability for Fe­(III) to increase the fluorescence of RBF and for CORM-3 to decrease the fluorescence of the RBF-Fe­(III) system, but not the concentration dependency of the system toward CORM-3. Overall, our new findings indicate that the RBF-Fe­(III) system does not sense CO. First, the system did not respond to CO gas bubbled into the RBF-Fe­(III) solution. Second, the original procedure described the use of physical agitation, which turned out to be the reason for the observed fluorescence changes regardless of whether the RBF-Fe­(III) solution was exposed to CO, N_2_, or under vacuum. Furthermore, many other factors also induced fluorescence decrease of the RBF-Fe­(III) system including vitamin C, acetate, and cysteine. All in all, the RBF-Fe­(III) system is sensitive to CORM-3, vitamin C, cysteine, sodium acetate and physical agitation. It does not sense CO and cannot be used in cell culture experiments for detecting CO. Interestingly, in the original publication, the RBF-Fe­(III) mixture was loaded into test strips for CO sensing work. However, because of a lack of reproducibility of the solution-phase results and a lack of chemistry reasons for the RBF-Fe­(III) complex to sense CO, we did not do further exhaustive studies to examine every aspect of the original paper.

In the end, we should emphasize the need to examine the basic chemistry principles in designing reaction-based CO probes or probes for other analytes. Furthermore, future reports should refrain from using CO and CORM interchangeably in experimental descriptions or results discussions, as if they represent the same thing; they are totally different. One should avoid using CO donors with intractable chemical reactivity in CO probe work and in studying CO biology. Looking back at many of the issues with fluorescent probes that ended up detecting only the donors, but not CO, we attribute these to the evolutionary nature of science and a historical snowballing effect, instead of the fault of a single lab. We all have to build our research on earlier work. When research becomes so interdisciplinary, it is hard to expect researchers to cross disciplinary boundaries to evaluate all the experimental details of earlier papers, such as in the case of using commercially available CORM-2 and CORM-3, which were specifically named “CO-releasing Molecules.” Therefore, we view our work related to metal/boron-based CORMs as a way to prevent future confusions and to put the CO research field into a healthy path and trajectory, not to assign blames or to criticize individual work. However, it is also important to emphasize the need to collectively correct historical mistakes and move on once the problems are clearly identified.

## Supplementary Material



## Data Availability

The authors declare that all data supporting the findings of this study are included within the article and its Supporting Information files. Raw data in alternative formats are available from the corresponding author upon reasonable request.

## Notes

Material and Instruments; experimental procedure; supporting figures; and NMR data (PDF)
