# Supplementary material for: An Fe(III)-Based Fluorescent Probe for Carbon Monoxide only Senses the “CO Donor” Used, CORM-3, but Not CO
Source: Anal Chem. 2025 Oct 9;97(41):22871–7. doi: 10.1021/acs.analchem.5c04712 (PMC12547852; doi:10.1021/acs.analchem.5c04712)
Supplement: Supplementary file 1 [file ac5c04712_si_001.pdf]

An Fe(III)-Based Fluorescent Probe for Carbon Monoxide only Senses the "CO Donor" Used, CORM-3, but Not CO

Hongliang Li,[#] Dongning Liu,[#] and Binghe Wang*

Department of Chemistry and Center for Diagnostics and Therapeutics, Georgia State University, Atlanta, Georgia 30303, United States

*Email: bwang31@gsu.edu.

[#]These two authors made equal contributions and are listed alphabetically.

Content

Experimental Section S2

Supporting Figures S3

References S8

Experimental Section

Material and Instruments

Chemical reagents were purchased from Sigma-Aldrich (Saint Louis, MO) and/or Oakwood (Estill, SC). Solvents were purchased from Fisher Scientific (Pittsburgh, PA). Dry solvents were prepared by a Vigor Tech purification system (Houston, TX). Certified pure CO calibration gas was purchased from GASCO (Oldsmar, FL). UV−vis absorption spectra were obtained by using a Shimadzu PharmaSpec UV-1700 UV−visible spectrophotometer (Kyoto, Japan). Fluorescence spectra were recorded on a Shimadzu RF5301PC fluorometer (Kyoto, Japan). ^{1}H NMR (400 MHz) and ^{13}C NMR (101 MHz) were acquired on a Bruker AV-400 MHz Ultra Shield NMR.

Synthesis of the RBF

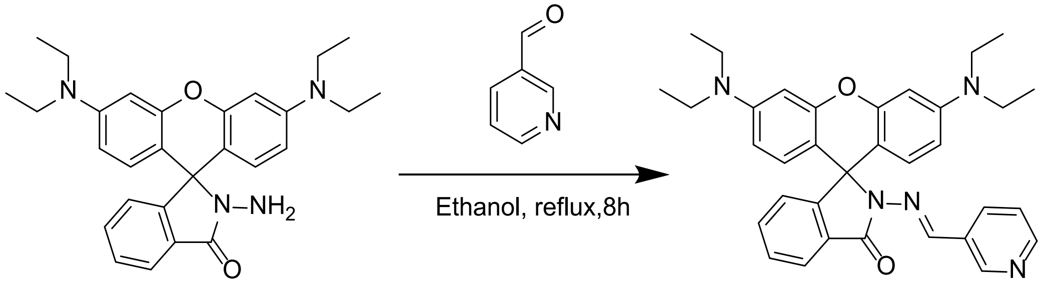

Scheme S1. Synthesis of RBF.

RBF was synthesized following a literature procedure.[1] Starting material (2.28 g, 5 mmol) was dissolved in 100 mL anhydrous ethanol followed by the addition of 3-pyridinecarboxaldehyde (0.65 g, 6 mmol). The mixture was heated under reflux for 8 hours and then cooled to room temperature. The reaction mixture was poured into 500 mL of water and left to stand for 8 hours. Afterward, the mixture was suction-filtered, and the residue was washed with water three times to obtain RBF (1.85g, 68%). ^{1}H NMR (DMSO) δ 9.09 (s, 1H), 8.55 (s, 1H), 8.49 (d, *J* = 3.0 Hz, 1H), 7.91 (s, 1H), 7.77 (d, *J* = 7.9 Hz, 1H), 7.58 (s, 2H), 7.34 (dd, *J* = 7.7, 4.8 Hz, 1H), 7.13 (d, *J* = 7.3 Hz, 1H), 6.42 (dd, *J* = 14.4, 5.5 Hz, 4H), 6.31 (dd, *J* = 8.8, 2.1 Hz, 2H), 3.28 (dd, *J* = 13.5, 6.6 Hz, 9H), 1.04 (t, *J* = 6.9 Hz, 13H). ^{13}C NMR (DMSO) δ 163.9, 152.9, 150.8, 148.5, 148.3, 145.5, 134.1, 133.2, 129.1, 127.8, 124.1, 123.2, 108.0), 105.6, 97.3, 65.9, 43.7, 12.4.

HR-MS (ESI): calcd for $C_{34}H_{36}N_5O_2$ (M + H^+): 546.2869; found 546.2875.

Experimental Procedure:

Fluorescence Response of RBF-Fe^{3+} Complex to bubbling CO gas

1 mL of the RBF-Fe(III) complex (10 μM) was placed in the fluorescence cuvette, pure CO gas was directly bubbled into the cuvette with a moderate flow rate through a long syringe needle (the pressure of CO gas tank is 10 psi) for 18 mins. Subsequently, fluorescence data were recorded.

Fluorescence Response of RBF-Fe^{3+} Complex to stir under Different Atmospheric Conditions.

The RBF-Fe(III) complex (10 μM) was placed in the round-bottom flask, which was then subjected to three cycles of evacuation and refilling with carbon monoxide (CO) gas to ensure an oxygen-free environment. The system was maintained under a continuous CO atmosphere, and the mixture was stirred at 800 rpm for 1 min or 18 mins. Subsequently, fluorescence measurements were carried out.

Supporting Figures

NMR spectra

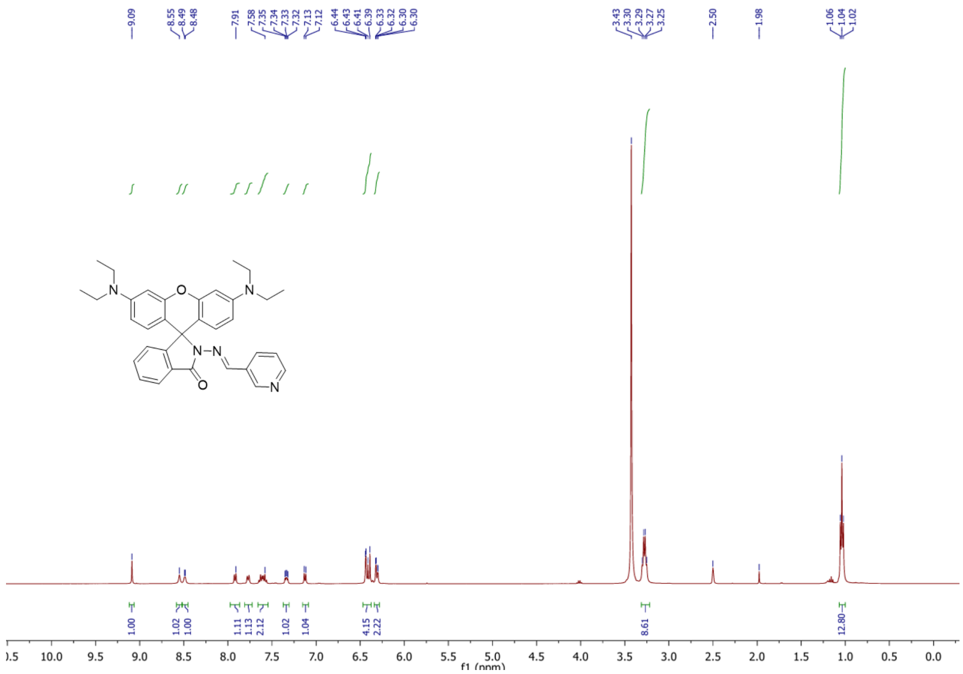

Figure S1. ^1H NMR spectrum of RBF in DMSO-d_6.

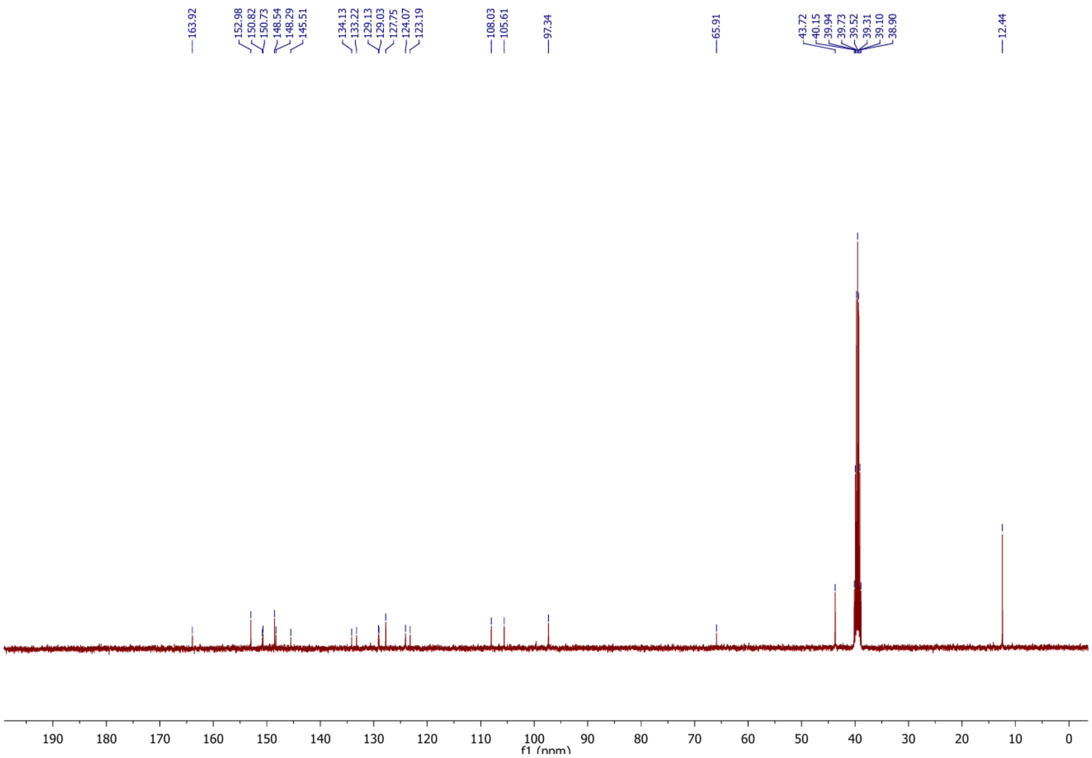

Figure S2. ^{13}C NMR spectrum of RBF in DMSO-d_6.

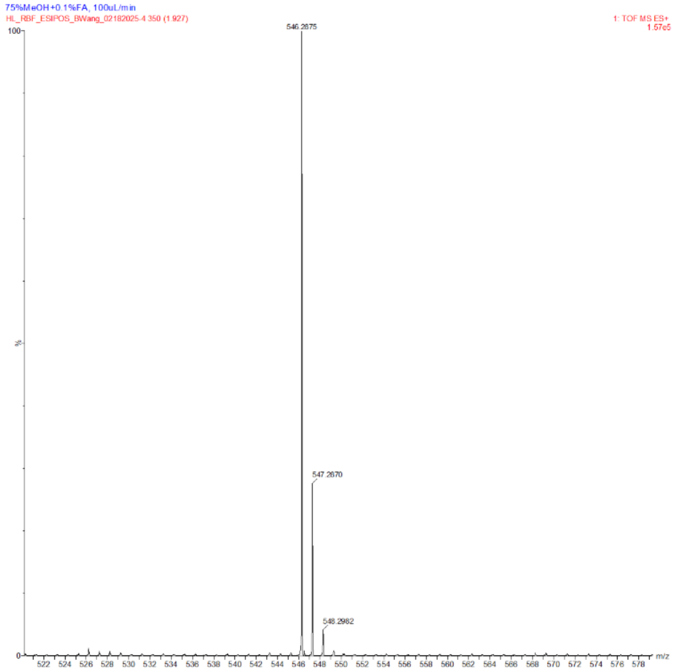

Figure S3. HRMS (ESI+) spectrum of RBF

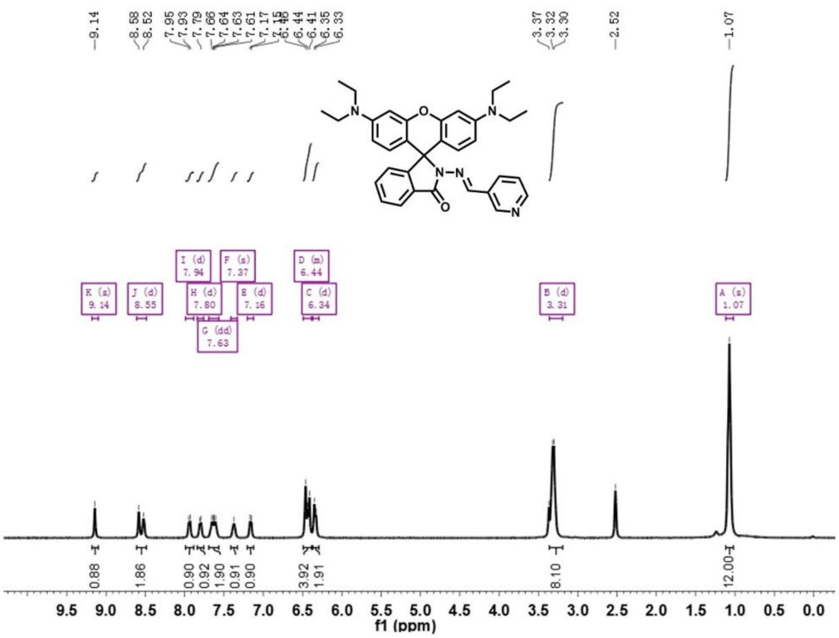

Figure S4. ^{1}H NMR spectrum of RBF in original literature. Reproduced with permission from Ref. [1]

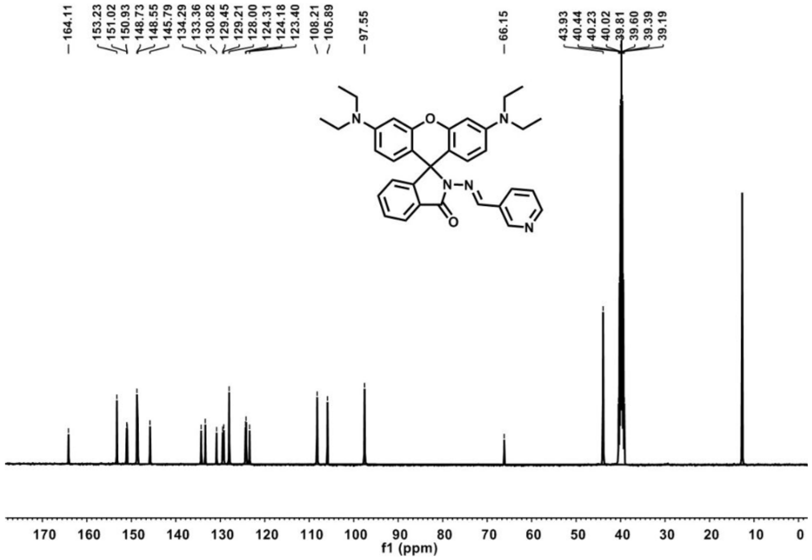

Figure S5. ^{13}C NMR spectrum of RBF reproduced from the original publication. Reproduced with permission from Ref.[1]

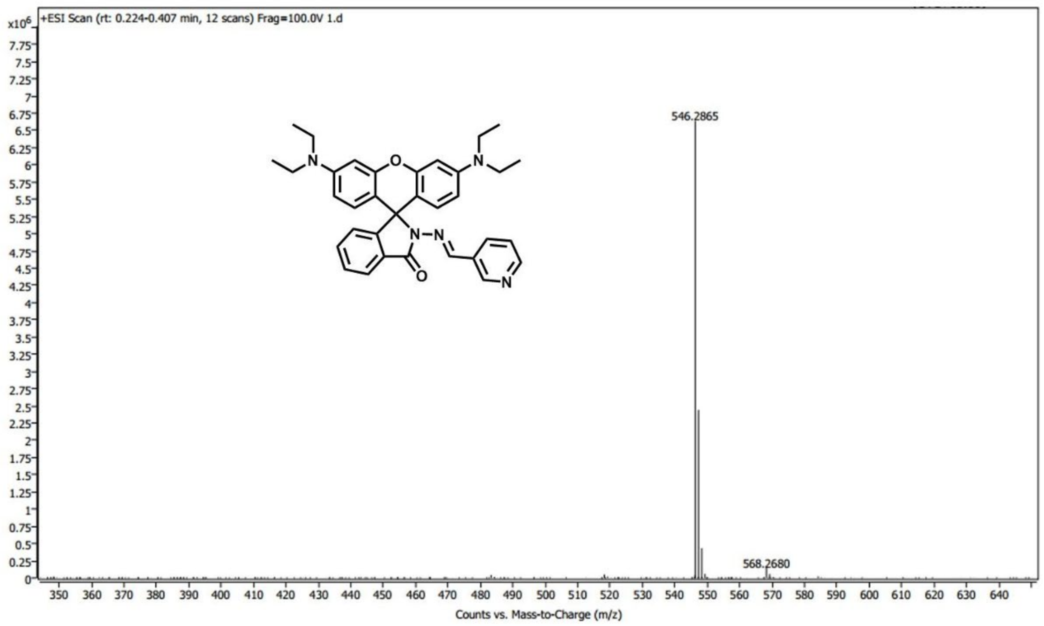

Figure S6. HRMS spectrum of RBF reproduced from the original publication Reproduced with permission from Ref.[1]

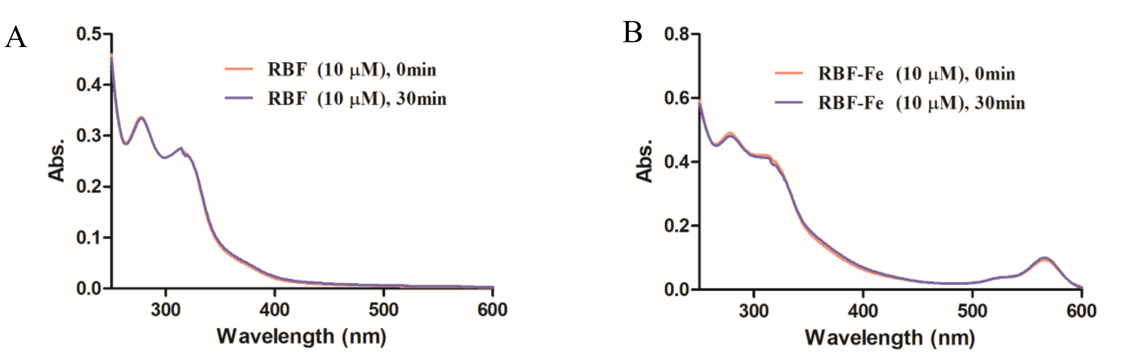

Figure S7. UV–Vis absorption spectra of **(A)** RBF (10 μM) and **(B)** its Fe(III) complex (RBF + $FeCl_3$, 5 equiv) measured at 1 min, and 30 min after mixing. All solutions were prepared in a DMSO/H_2O solvent system (v/v = 1:4) and maintained under static conditions. The overlapping spectra confirm that both RBF and the RBF-Fe complex are stable over time in the absence of external disturbance.

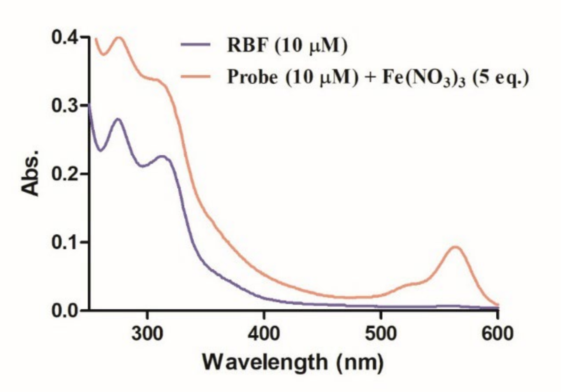

Figure S8. UV–Vis absorption spectra of RBF (10 μM) after addition of $Fe(NO_3)_3$. Characteristic absorbance peak appears around 564 nm upon addition of Fe^{3+} salts.

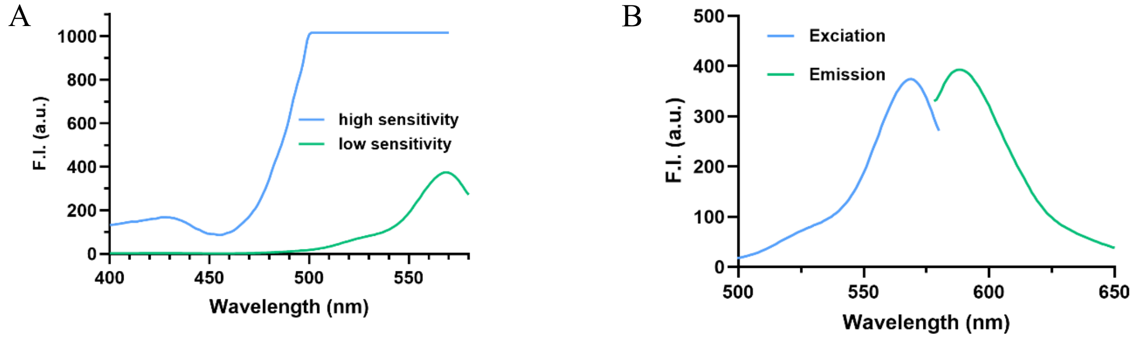

Figure S9. **(A)** Excitation spectrum for RBF-Fe complex (RBF-10 μM, $FeCl_3$ 50 μM) in DMSO/H_2O (v/v = 1:4) with two different sensitivity settings. (λ_{em} = 590 nm). **(B)** Excitation and emission spectra for RBF-Fe complex (RBF-10 μM, $FeCl_3$ 50 μM) in DMSO/H_2O (v/v = 1:4). (λ_{ex} = 570 nm, λ_{em} = 590 nm, slit width = 5 nm)

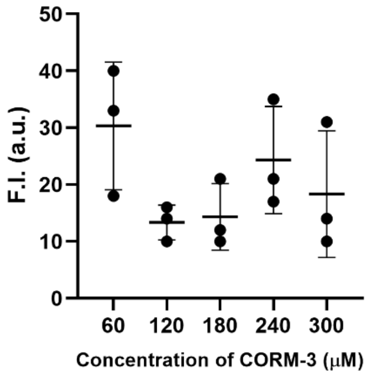

Figure S10. Effects of CORM-3 (60-300 μM in DMSO and H_2O) on the fluorescence of RBF-Fe complex (RBF-10 μM, $FeCl_3$ 50 μM) in DMSO/H_2O (v/v = 1:4). A bar figure for triplicate results from Figure S10A. (Ex = 450 nm, λ_{em} = 590 nm, slit width = 5 nm)

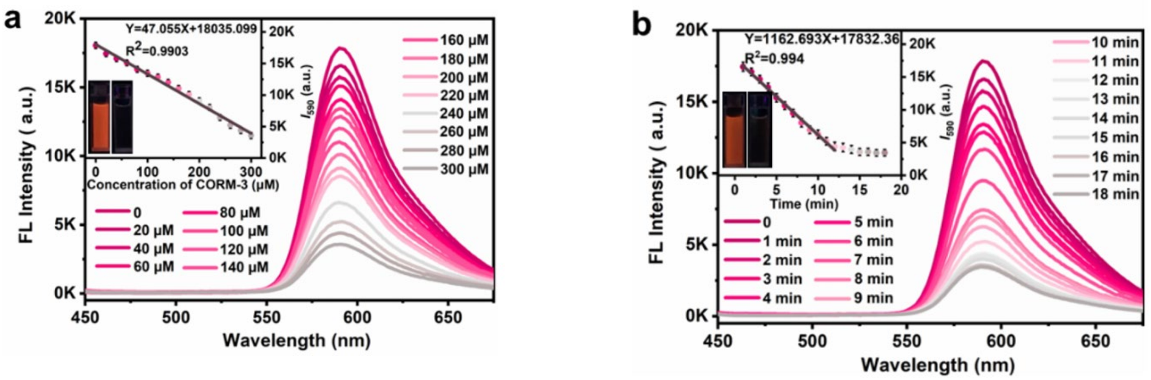

Figure S11. (a) Fluorescence spectra of RBF-Fe (10 μM) in a DMSO−water (1:4) solvent mixture upon addition of varying concentrations of CORM-3. *Inset:* linearity of fluorescence intensity with respect to CORM-3 concentrations. (b) Fluorescence spectra of RBF-Fe (10 μM) in a DMSO−water (1:4) solvent mixture upon exposure to CO for 0−18 min. *Inset:* linearity of fluorescence intensity with respect to CO exposure time. Figures reproduced permission from the original publication.[1]

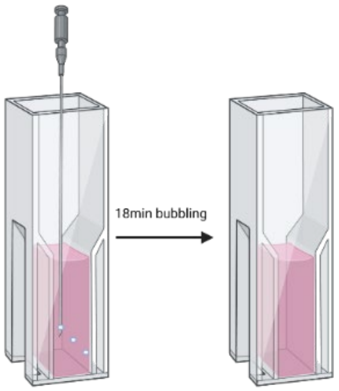

Figure S12. Schematic representation of CO bubbling into the RBF-Fe(III) solution (10 μM) using a narrow-bore connector to minimize bubble diameter. This setup is designed to reduce disturbance to the solution during CO delivery.

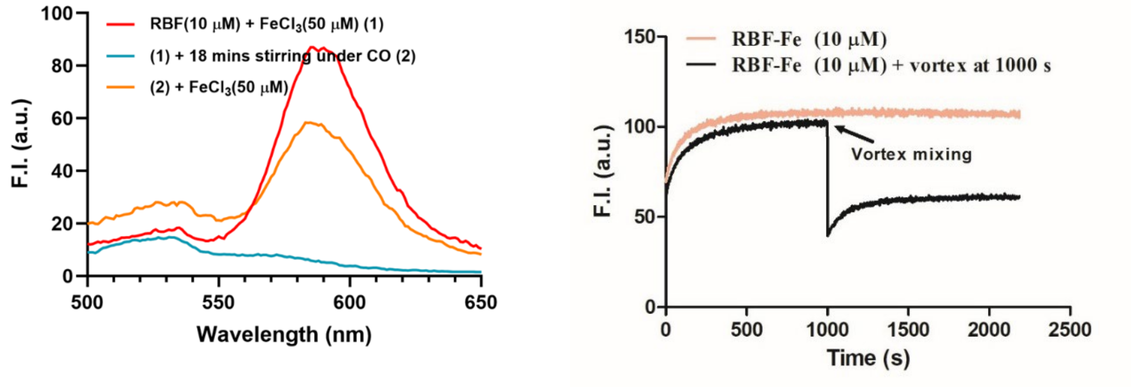

Figure S13. A. Fluorescence spectra of RBF-Fe complex (RBF-10 μM, $FeCl_3$ 50 μM) after stirring at 800 rpm for 18 minutes at room temperature under pure CO gas, followed by adding 50 μM $FeCl_3$. B. Time-course fluorescence response of RBF-Fe complex (RBF-10 μM, $FeCl_3$ 50 μM) monitored at 590 nm. The fluorescence intensity increased gradually over time, reaching a plateau before 1000 seconds. At t = 1000 s, vortex mixing was applied to the solution for 30 seconds. (Ex = 450 nm, λ_{em} = 590 nm, slit width = 5 nm)

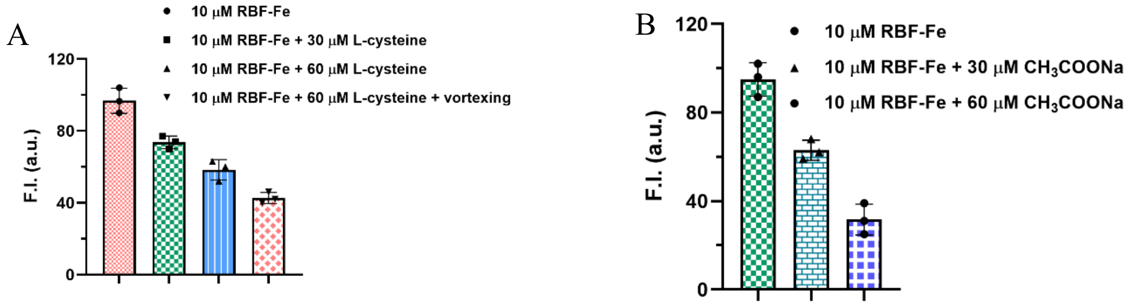

Figure S14. Fluorescence intensity changes of RBF-Fe complex (RBF-10 μM, $FeCl_3$ 50 μM) after addition of **(A)** L-cysteine with a final concentration of 30 μM, 60 μM, and vortexing after 60 μM L-cysteine **(B)** sodium acetate with a final concentration of 30 μM and 60 μM. (Ex = 450 nm, λ_{em} = 590 nm, slit width = 5 nm)

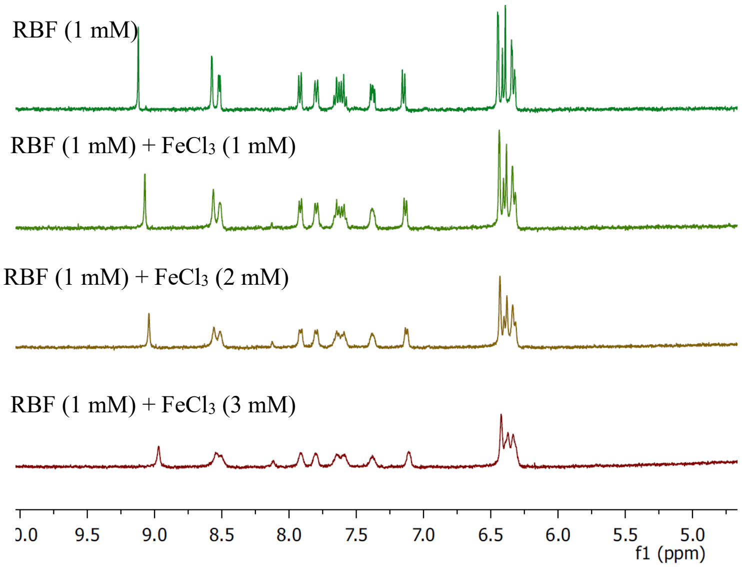

Figure S15. ^{1}H NMR spectra for RBF (1 mM) with $FeCl_3$ (1-3 mM) in DMSO-d_6 (588 μL) and D_2O (12 μL).

Reference

1. Fang, X.; Cui, L.; Yu, H.; Qi, Y. Fe(III)-Based Fluorescent Probe for High-Performance Recognition, Test Strip Analysis, and Cell Imaging of Carbon Monoxide. *Anal. Chem.* **2024,** *96* (28), 11588–11594.
